# Supplementary material for: Ratiometric nanothermometer in vivo based on triplet sensitized upconversion
Source: Nat Commun. 2018 Jul 12;9:2698. doi: 10.1038/s41467-018-05160-1 (PMC6043590; doi:10.1038/s41467-018-05160-1)
Supplement: Supplementary file 1 — Supplementary Information [file 41467_2018_5160_MOESM1_ESM.pdf]

Supporting Information

Ratiometric Nanothermometer *in Vivo* Based on Triplet-Sensitized Upconversion

Xu *et al.*

Ratiometric Nanothermometer *in Vivo* Based on Triplet-Sensitized Upconversion

Ming Xu, Xianmei Zou, Qianqian Su, Wei Yuan, Cong Cao, Qiuhong Wang, Xingjun Zhu, Wei Feng* and Fuyou Li*

Department of Chemistry & State Key Laboratory of Molecular Engineering of Polymers, Fudan University, Shanghai 200433, P. R. China.

Supplementary Methods

Materials. All reagents and solvents were used as received without further purification unless otherwise indicated. Pt complex tetraphenyltetrabenzoporphyrin (PtTPBP, the sensitizer of TTA system) was purchased from Luminescence Technology Crop. Soybean oil, oleic acid and 1-octadecene were purchased from Sigma-Aldrich Co., LLC. Analytical grade toluene, methanol, ethanol, cyclohexane, hydrochloric acid, NaOH and NH_4F were purchased from Sinopharm Chemical Reagent Co., Ltd. The rare-earth oxides Ln_2O_3 (purity: 99.999%) (Ln=Y, Nd) were purchased from Beijing Lansu Co. Rare-earth chlorides were prepared by dissolving the corresponding metal oxide in hydrochloric acid at elevated temperature. Paraffin liquid (nujol) and sodium phosphotungstate were purchased from Acros. Bovine serum albumin (BSA, molecular biology grade) was purchased from the Aladdin Industrial Co., Ltd. L-Ascorbyl 6-palmitate was purchased from J&K Scientific Ltd. Deionized water was used was used throughout the experiments. Compounds 2,6-diethyl-1,3,5,7-tetramethyl-8-Trimethylphenyl-4,4-difluoroboradiazaindacene (BDM, the annihilator of TTA system) and Nd^{3+} nanophosphors were synthesized according to the previous literatures with some modifications.[S1,S2]

Characterization. UV-vis absorption spectra were measured with an UV-vis spectrophotometer (Schimadzu, UV-2550). ^{1}H-NMR spectra were measured with Bruker spectrometer at 400MHz (AVANCE III). X-ray powder diffraction

(XRD) measurement was performed on a diffractometer with graphite monochromated Cu Ka radiation (Bruker D4). The morphology of nanoparticles were determined by a transmission electron microscope (TEM, JEOL JEM-2010). The emission spectra were recorded with a fluorescence spectrometer (Edinburgh, FLS-920). For the measurement of UCL (or NIR) emission spectra, an external continuous-wave (CW) 635 nm (or 808 nm) semiconductor laser (Changchun fs-optics Co., China) was used as the excitation source, instead of the Xeon lamp in the spectrophotometer. In the lifetime measurements, a tunable continuous wavelength pulse laser (opotek Inc., opolette 355) was used as excitation source. The absolutely quantum efficiency was measured with the Hamamatsu instrument (C13532-12 Quantaurus-QY plus). Dynamic laser scattering and zeta-potential measurements were carried out on a commercial Zetasizer Nano instrument (Malvern, ZS90). The IR thermal camera (FLIR E40) was used to imaging the temperature distributions on the surface of the mice.

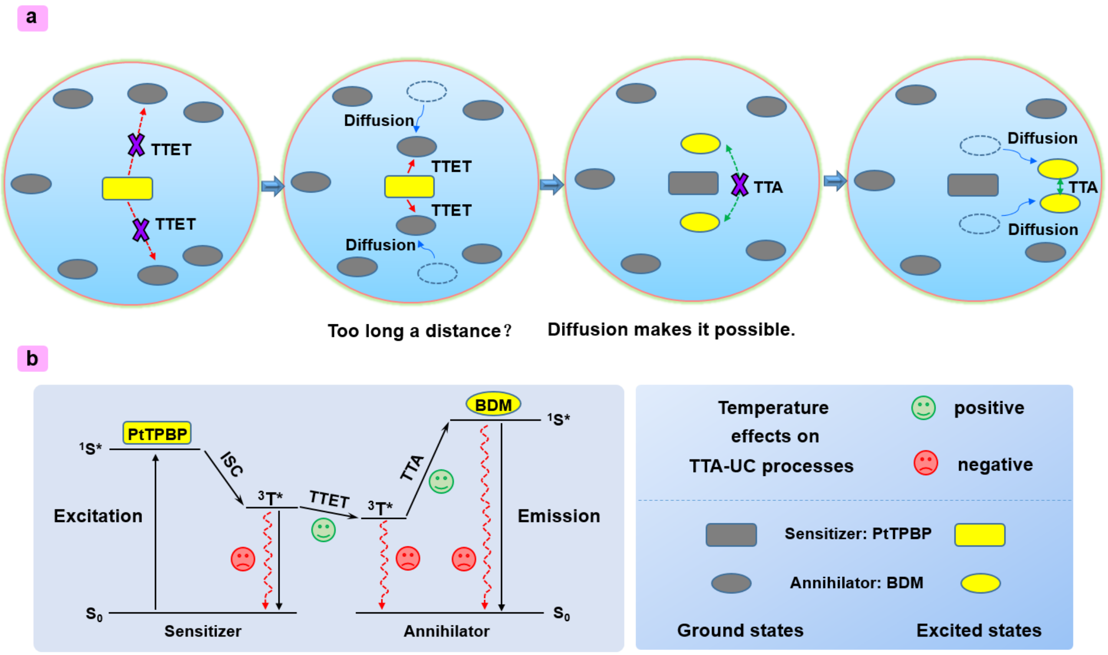

Supplementary Figure 1. The TTA-upconversion mechanism in related to temperature response elaborated in two different ways. Energy transfer (such as TTET and TTA process) between two chromophores far apart is impossible. Diffusion makes it possible. Consequently, the diffusion of chromophores (positively correlated with temperature) is a positive effect for the eventual TTA-UCL emission (a). However, the nonradiative transition of chromophores (positively correlated with temperature) is a negative effect for the eventual TTA-UCL emission (b). Therefore, the suppression of nonradiative deactivation is beneficial to the positively thermalsensitive property in a TTA-UC system.

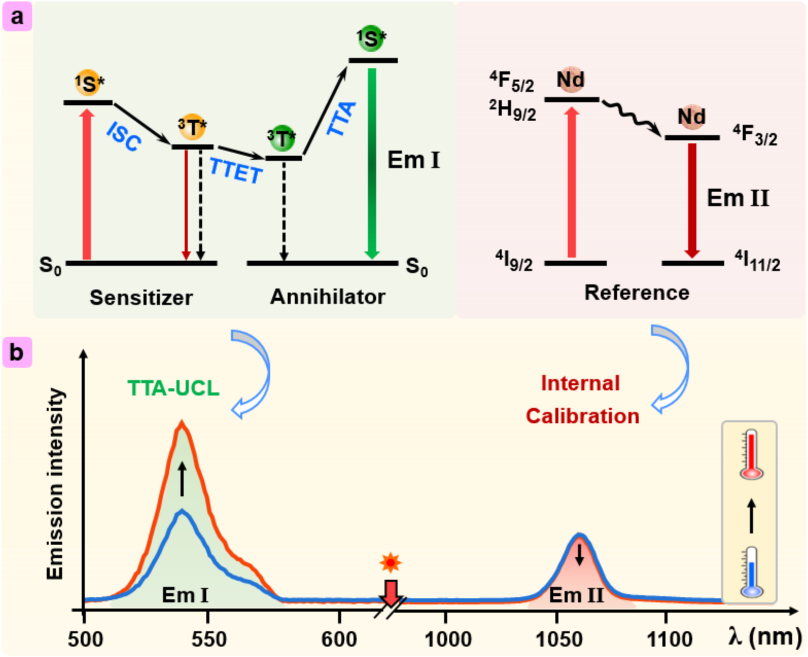

Supplementary Figure 2. The schematic energy transfer diagram (a) and the emission spectrum (b) of TTA-Nd-NPs under excitation at 635 & 808 nm. The two emission bands (Em I and Em II) were attributed to upconversion luminescence of TTA chromophores (BDM & PtTPBP) and NIR luminescence of Nd^{3+} nanophosphors, respectively. The calibration signal had minimized overlap with the TTA-UCL signal, which could avoid the crosstalk effect.

Supplementary Table 1. Experimental parameters of the *in vivo* nanothermometry reported in scientific researches.[S3] λ_{Ex}, I_{Ex}, λ_{Opr}, S_r and R_{Ther} correspond to excitation wavelength, excitation laser power density, operating wavelength, relative thermal sensitivity and thermal resolution, respectively.

Material	Size (nm)	λ_{Ex} (nm)	I_{Ex} (W cm^{-2})	λ_{Opr} (nm)	S_r (10^{-2} K^{-1})	R_{Ther} (K)
GFP[S4]	-	473	-	480-560	1.46	-
Pbs/CdS/ZnS QDs[S5]	6	808	0.1-3.0	850-1650	1.25	-
LaF_3:Nd^{3+} NPs[S6]	15	808	4	800-930	0.26	-
CsUCNP@C[S7]	77	730 and 980	0.3-0.8	500-580	1.10	0.5
Nd@Yb LaF_3 NPs[S8]	24	808	0.7	900-1360	0.4	1
TTA-Nd-NPs[this work]	165	635 and 808	0.1	540 and 1060	7.1	0.1

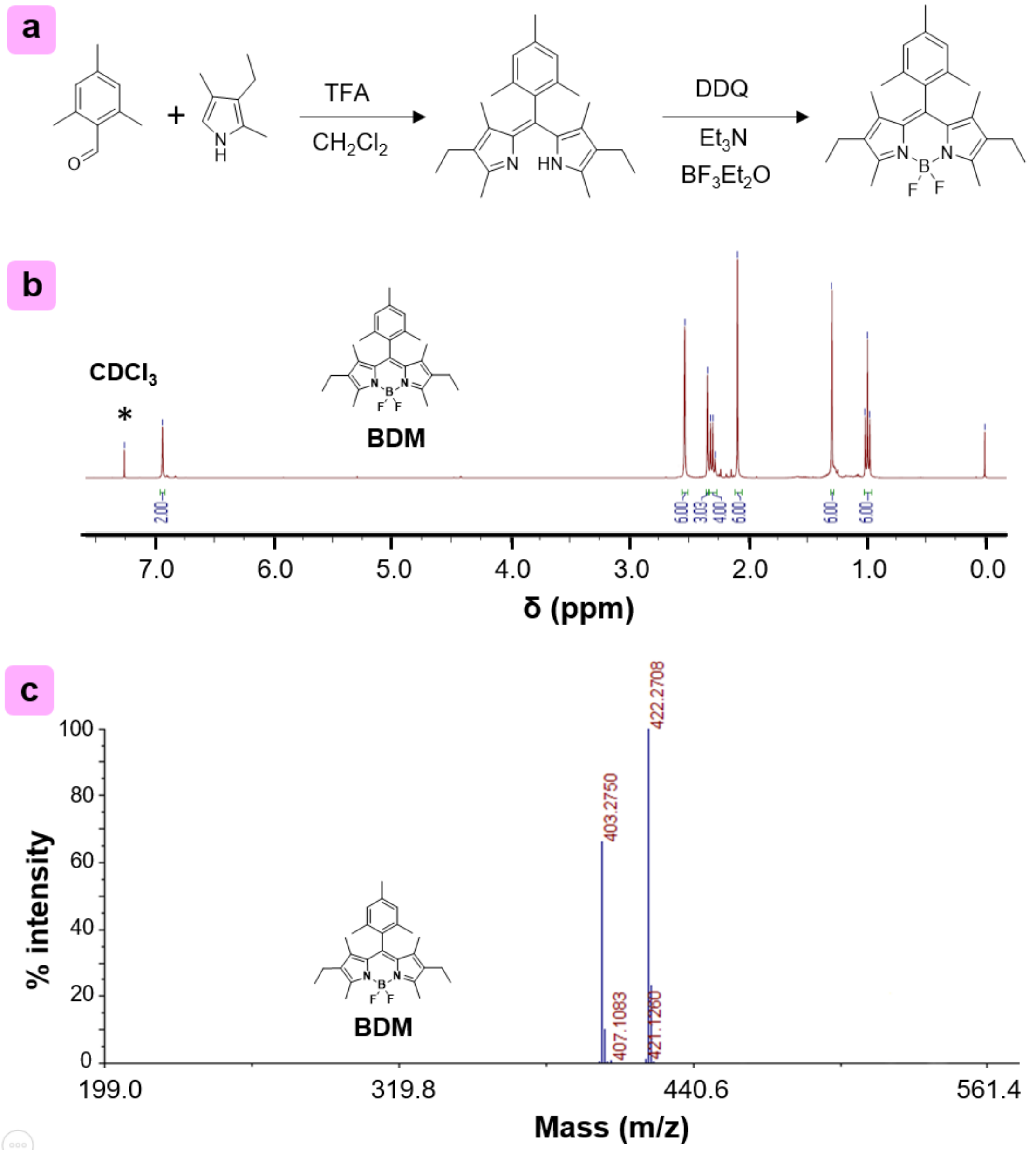

Supplementary Figure 3. The synthesis and characterization of BDM. a) The synthesis procedures of BDM. b) The ^{1}H-NMR spectrum of BDM in $CDCl_3$. c) The mass spectrum of BDM.

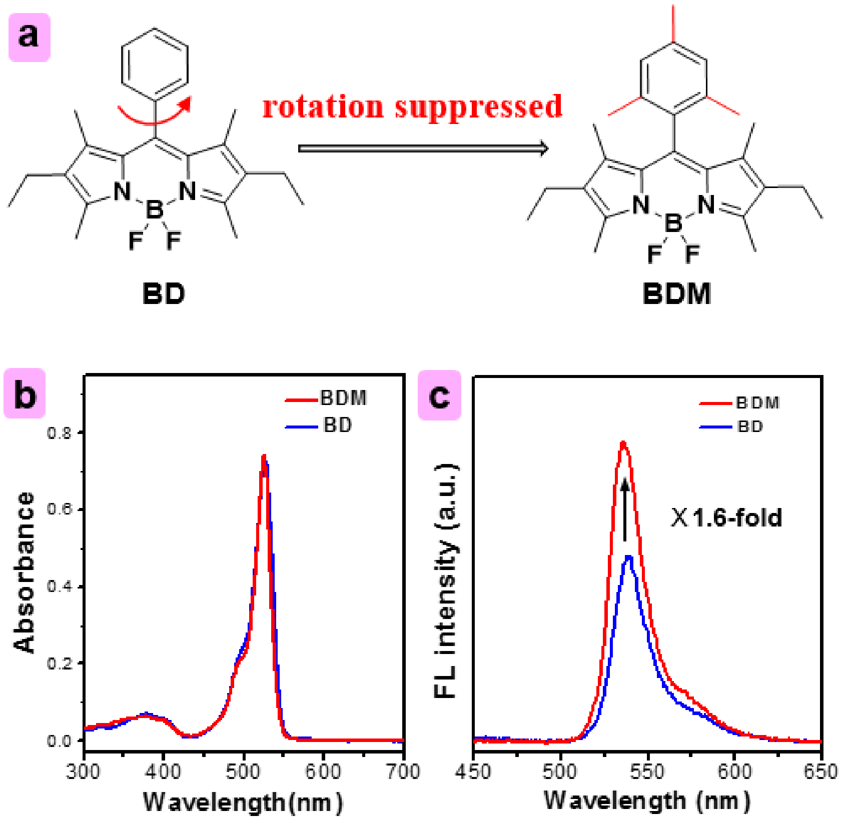

Supplementary Figure 4. The structure modification and thus enhanced performances. a) Methyl groups were added to the phenyl ring of a conventional BODIPY annihilator (BD) to suppress the free rotation of phenyl ring in BDM. b–c) The UV-vis absorption (b) and fluorescence spectra (c) of BD and BDM, respectively.

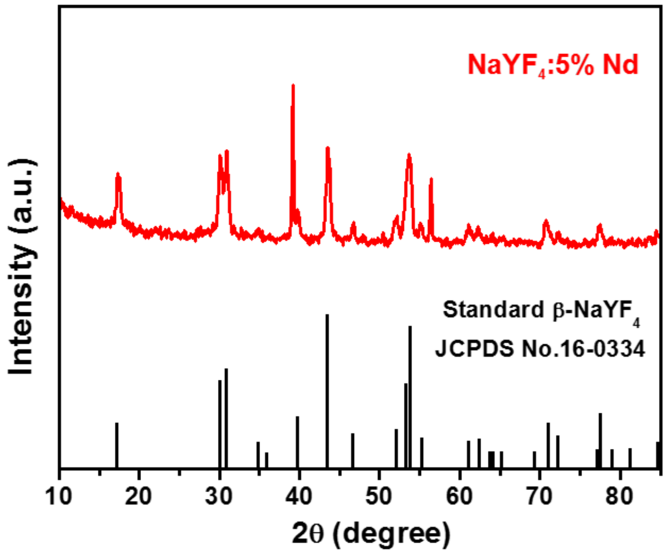

Supplementary Figure 5. The XRD pattern of the reference $NaYF_4$: 5% Nd nanophosphors.

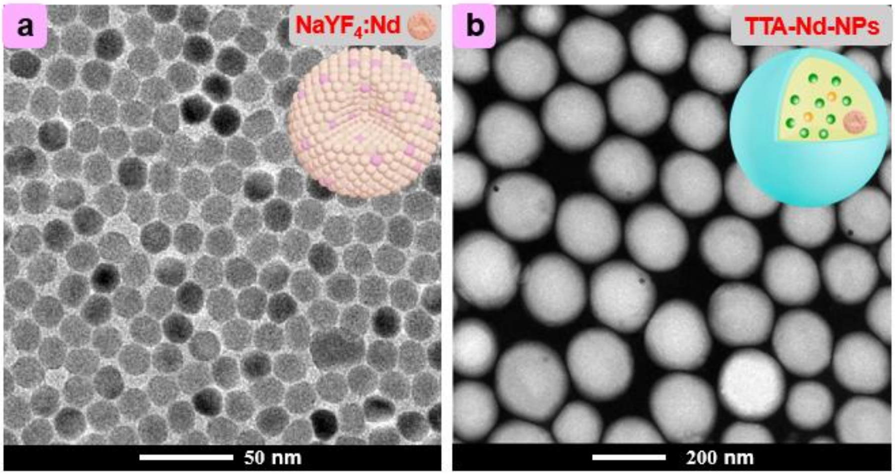

Supplementary Figure 6. The TEM characterization of the organic-inorganic TTA-Nd-NPs nanocomposite. a) The TEM image of the Nd^{3+} nanophosphors that was served as calibration unit. b) The TEM image of TTA-Nd-NPs after negative stain with sodium phosphotungstate.

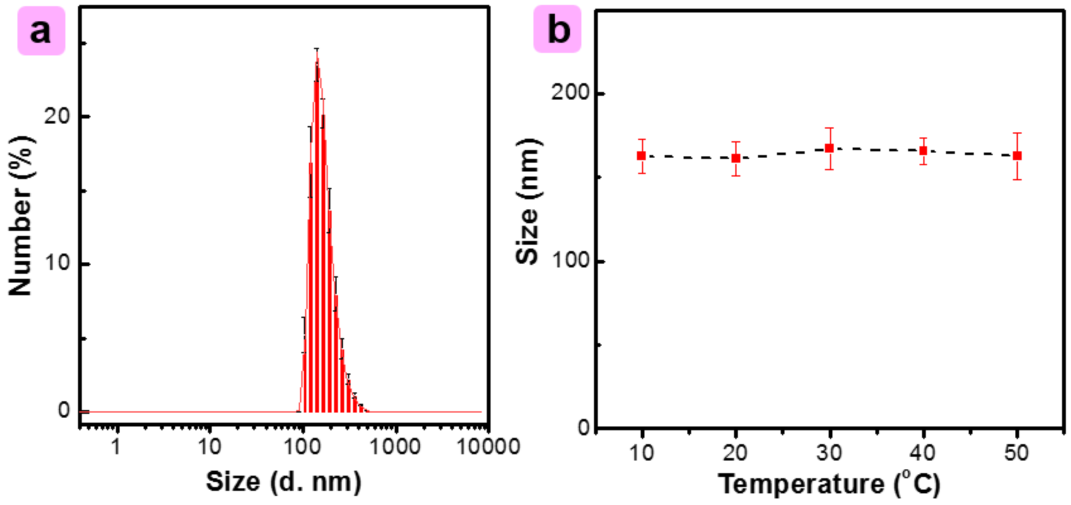

Supplementary Figure 7. a) The hydrodynamic diameters of the TTA-Nd-NPs as indicated in the dynamic light scattering measurement. b) The hydrodynamic size of TTA-Nd-NPs measured at different temperature in the range of 10–50 ˚C.

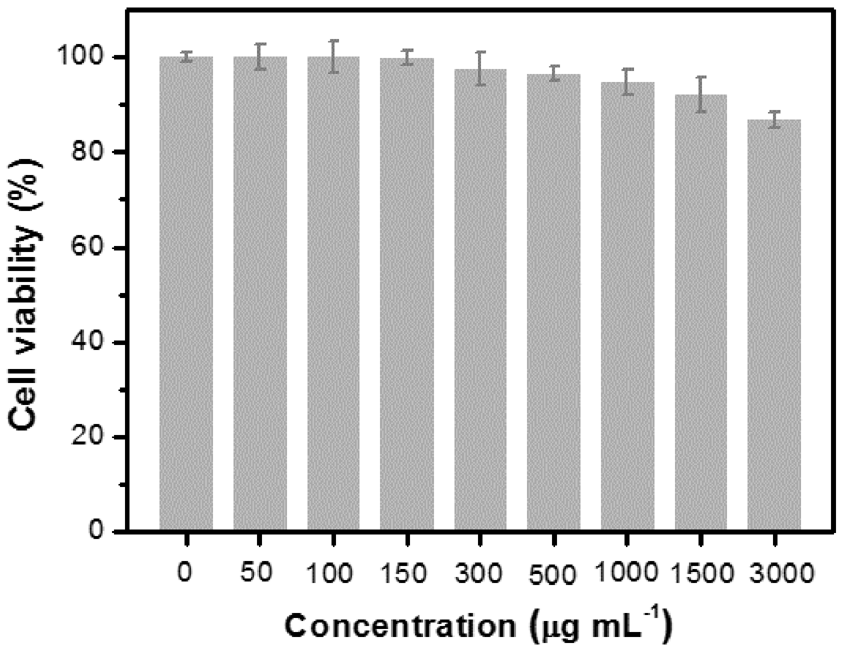

Supplementary Figure 8. The cell viability of Hela cells that were incubated with TTA-Nd-NPs (0.05–3.0 mg mL^{-1}) for 24 h.

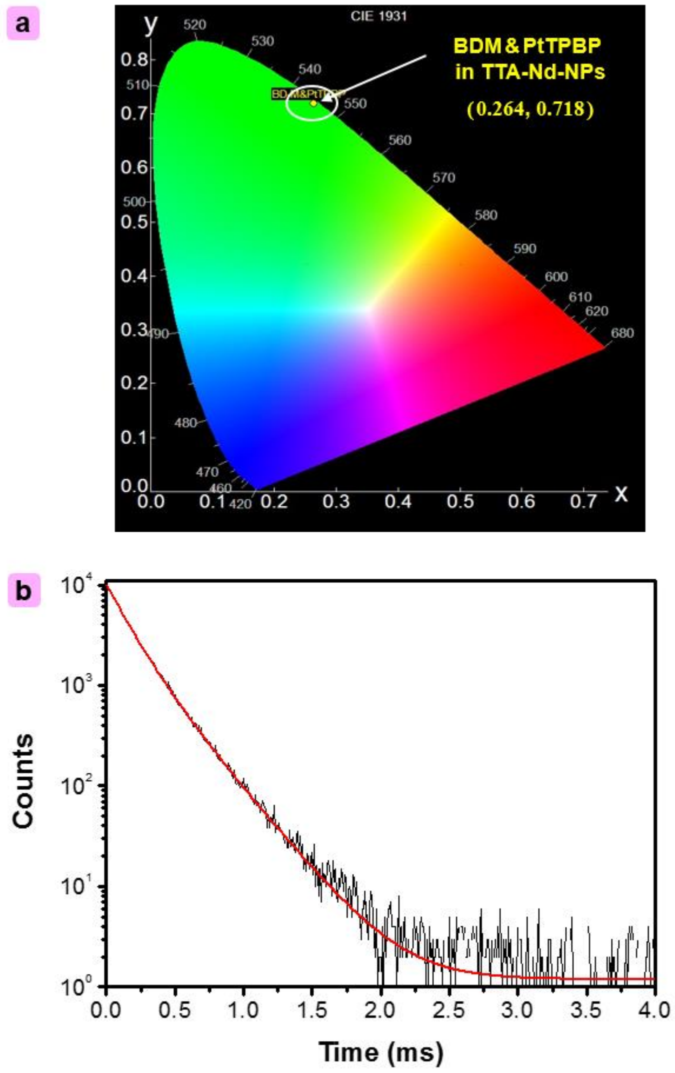

Supplementary Figure 9. a) The green upconversion luminescence of BDM & PtTPBP in TTA-Nd-NPs nanoplatform under excitation of 635 nm laser (100 mW cm^{-2}). The chromaticity coordinate of upconversion emission is (0.264, 0.718). b) The upconversion lifetime (τ_{UCL}) at 545 nm of TTA-Nd-NPs. A tunable continuous wavelength pulse laser at 635 nm was used as the excitation source. And a 630 nm short pass filter was used to cut off the laser light. τ_{UCL} = 170 μs.

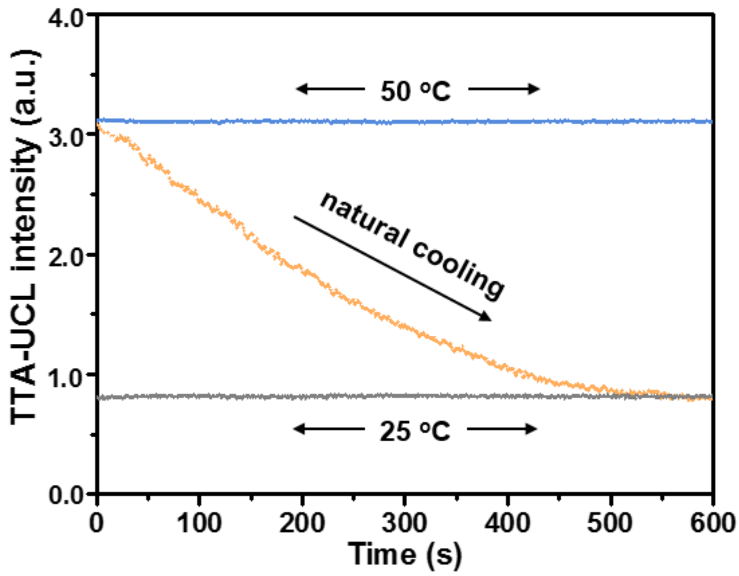

Supplementary Figure 10. The temperature response of TTA-UCL signal emitted from TTA-Nd-NPs that returned to room temperature from 50 °C through natural cooling process (yellow line). The control TTA-Nd-NPs samples were kept at 50 °C (blue line) and 25 °C (black line), respectively. The UCL intensities of theses identical samples were dynamically recorded on the fluorescence spectrometer under the same measurement conditions. Power density of the 635 nm excitation laser was 100 mW cm^{-2}.

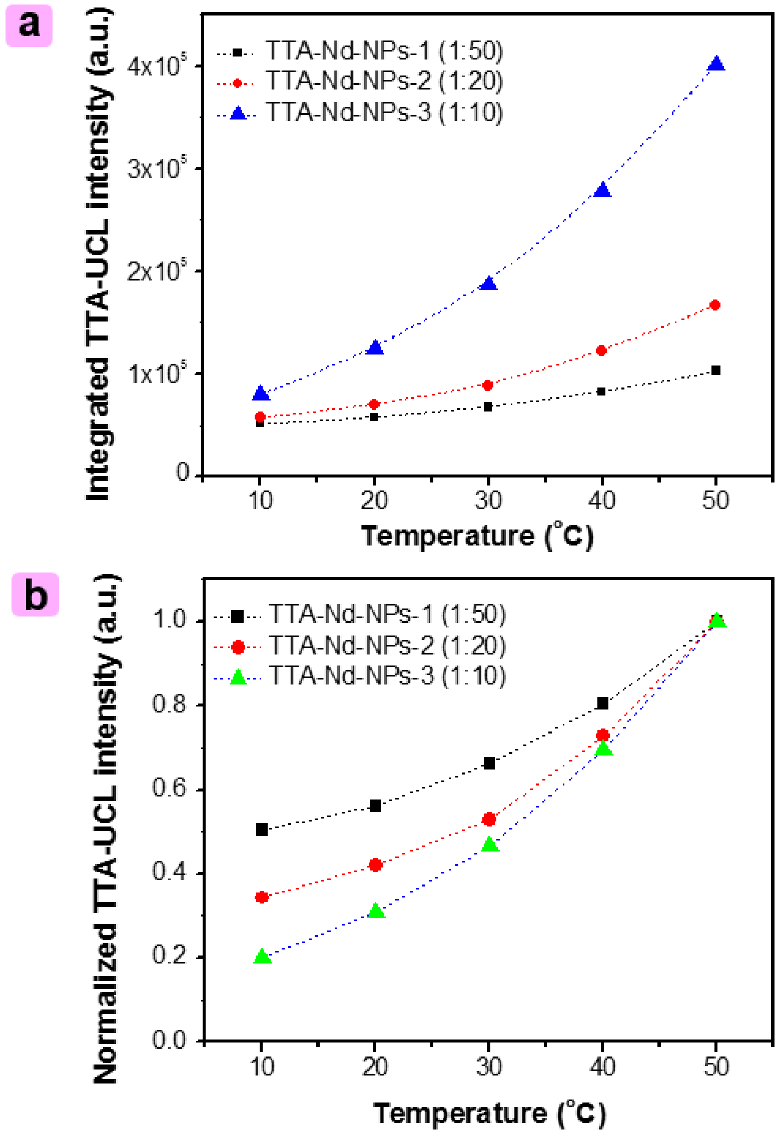

Supplementary Figure 11. a) The temperature dependence of TTA-Nd-NPs with different sensitizer-to-annihilator ratios. b) The maximum UCL intensities at 50 °C were all normalized to 1. In the preparation procedure, the annihilator (BDM) with a concentration of 2×10^{-3} M in nujol was used. And concentration of the sensitizer (PtTPBP) was 4×10^{-5} M, 1×10^{-4} M and 2×10^{-4} M in nujol for the preparation of TTA-Nd-NPs-1, TTA-Nd-NPs-2 and TTA-Nd-NPs-3, respectively.

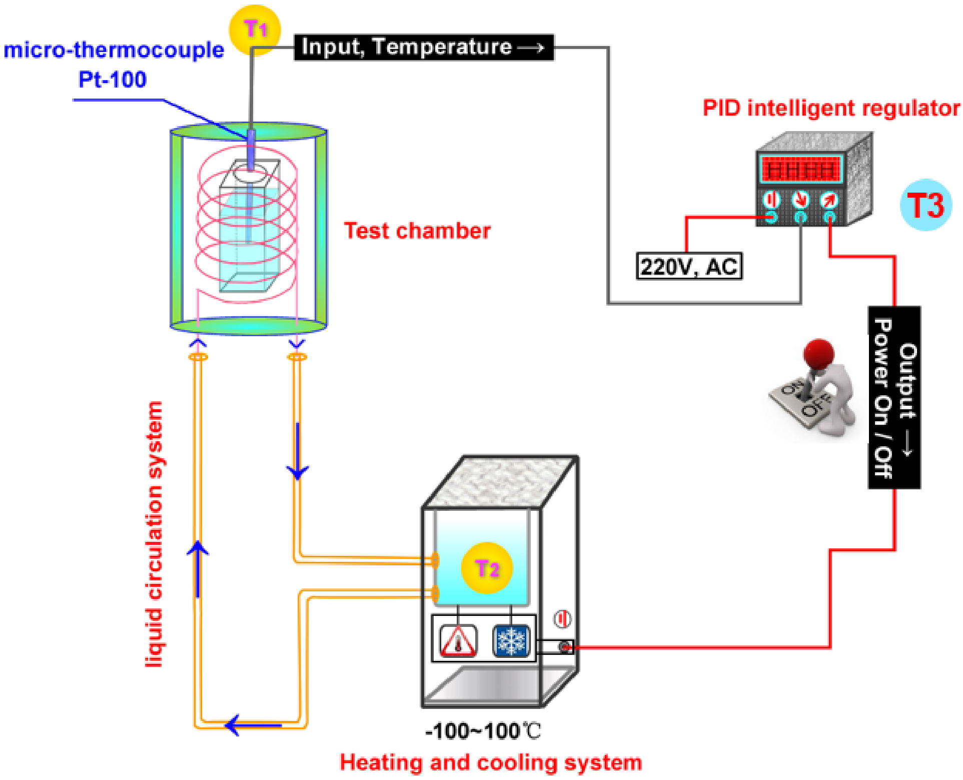

Supplementary Figure 12. Schematic illustration of the temperature controlling system designed for spectra measurements. Generally, temperature in the solution of sample (T1) was different from that in the heating and cooling system (T2). The intelligent proportion-integration-differentiation (PID) regulator with an assignable temperature (T3) would control the heating-cooling process until the input T1 is close to T3 (within limits of ±0.1 K). As a result, T1 could be regulated to the same value with T3. Therefore, the solution temperature of tested sample was easily controllable to reach a specified temperature.

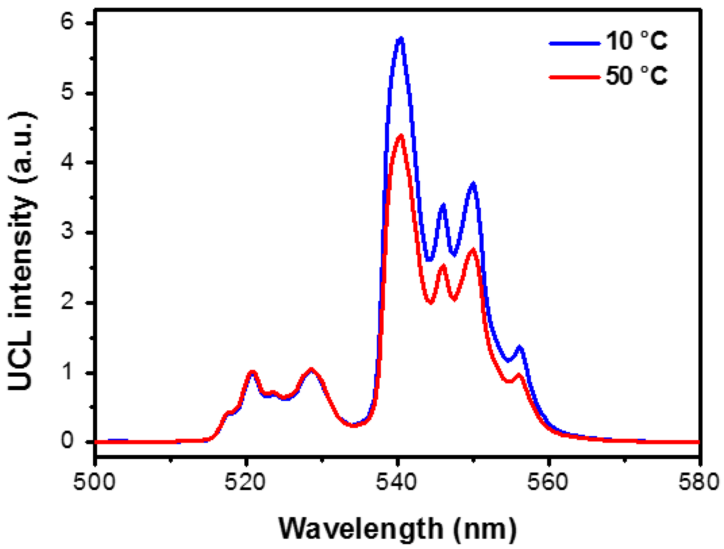

Supplementary Figure 13. The upconversion spectra of $NaYF_4$: Yb, Er @ $NaLuF_4$ core-shell nanophosphors under 980 nm laser excitation at 10 ^{o}C (blue line) and 50 ^{o}C (red line), respectively. The $NaYF_4$: Yb, Er @ $NaLuF_4$ material could serve as a representative Ln-UCNPs which had been developed for thermometry applications (*Nat. Commun.* **2016**, *7*, 10437).[s7]

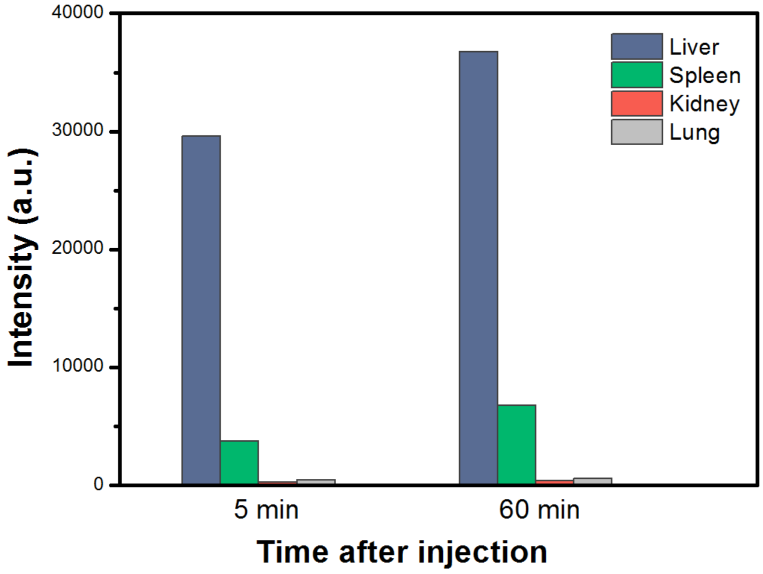

Supplementary Figure 14. TTA-UCL intensities of mice organs at various time points post-injection. The mice were intravenously injected with TTA-Nd-NPs. The emission signals were acquired in the TTA-upconversion channel I (485–575 nm) of the bioimaging system.

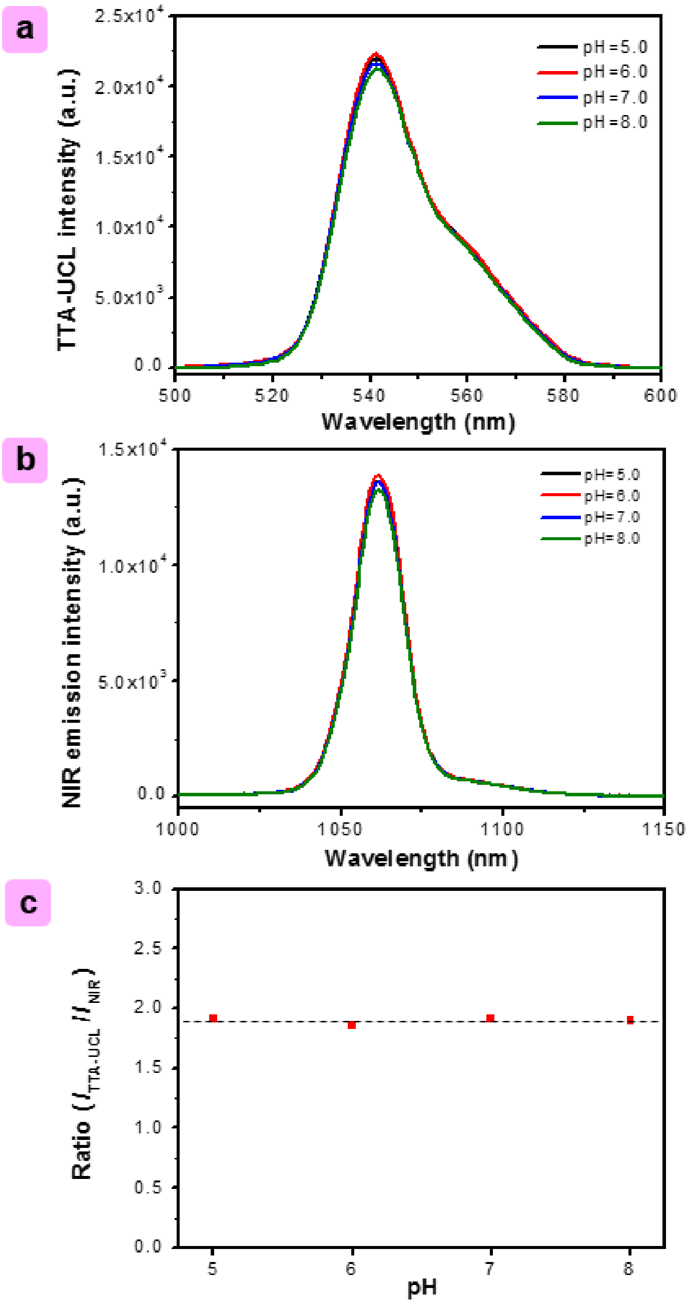

Supplementary Figure 15. The intensity variations of UCL emission from TTA-UCL a) and NIR emission from Nd^{3+} nanophosphors b) in TTA-Nd-NPs with different pH values in the range of 5.0–8.0. c) The Ratio of TTA-UCL emission to NIR emission at different pH values.

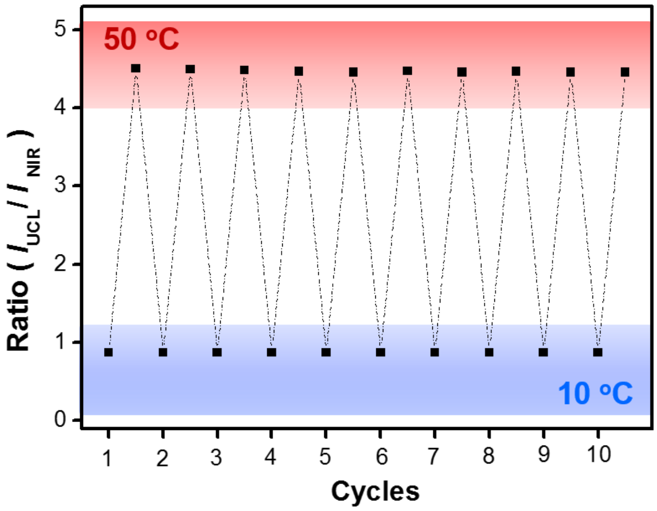

Supplementary Figure 16. The reversibility of TTA-Nd-NPs tested in continuous warming-cooling cycles. Ratiometric signals (I_{UCL}/I_{NIR}) were measured at 10 °C and 50 °C, respectively. I_{UCL}: intensities of TTA-UCL signal at 540 nm; I_{NIR}: intensities of Nd-NIR signal at 1060 nm.

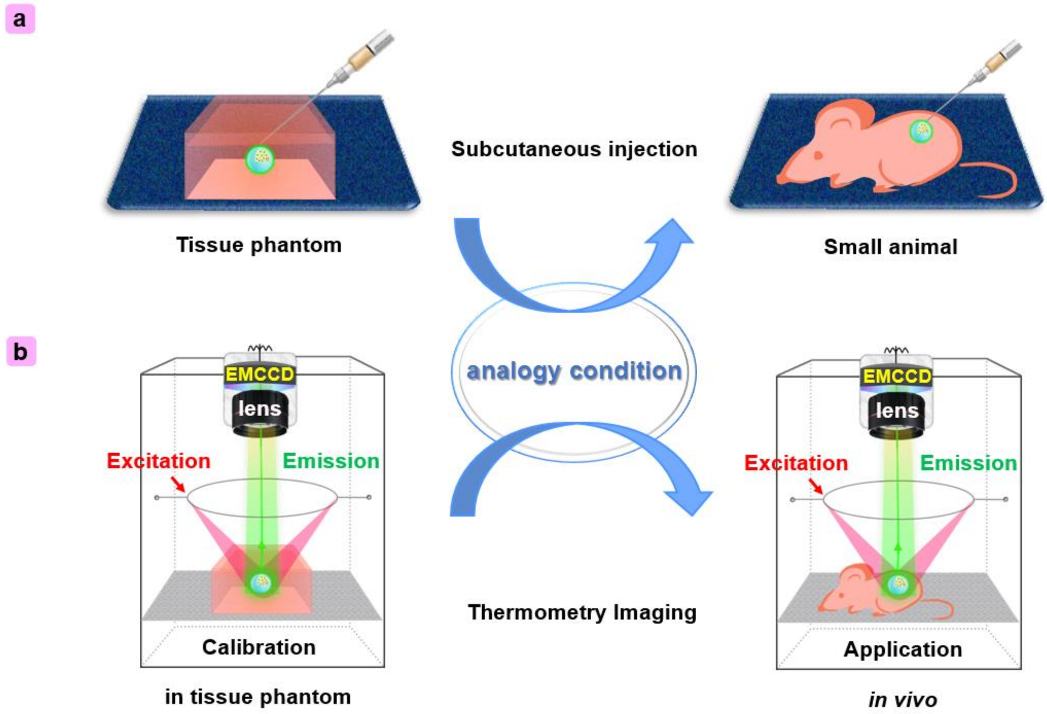

Supplementary Figure 17. The analogy conditions of the calibration situation and the practical application. In the phantom, haemoglobin and intralipid in gelatin were used to simulate absorption and scattering effects, respectively. Therefore, the phantom was similar with the biological tissue in optical property. The standard curve for temperature evaluation *in vivo* was measured with TTA-Nd-NPs probe in tissue phantom. The uniform condition of the injection (a) and imaging (b) methods could guarantee the accuracy for thermometry *in vivo*.

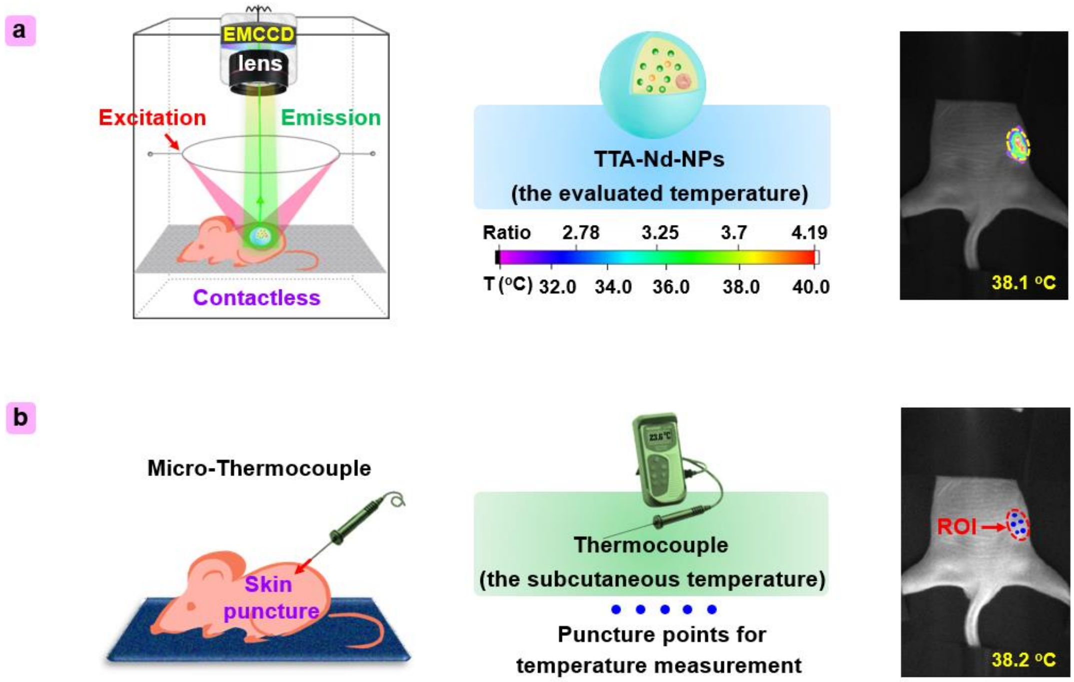

Supplementary Figure 18. Thermometry *in vivo* based on ratiometric probe TTA-Nd-NPs a) and micro-thermocouple b). a) The luminescent images in two different channels were acquired under the excitation of 635 & 808 nm laser: Channel I (485–575 nm, green emission band of TTA-UCL) and Channel II (980–1300 nm, NIR emission band of Nd^{3+} nanophosphors). The ratio ($I_{\text{Channel I}}$ / $I_{\text{Channel II}}$) and temperature distribution in regions of ROI were calculated according to the standard curve. b) The subcutaneous temperature was acquired with a micro-thermocouple. The thermocouple was punctured into the body to measure temperature under skin directly. And several puncture points were necessary to obtain the average temperature in a specific region. A small deviation of about 0.1 K in the average temperatures was observed for these two thermometry methods.

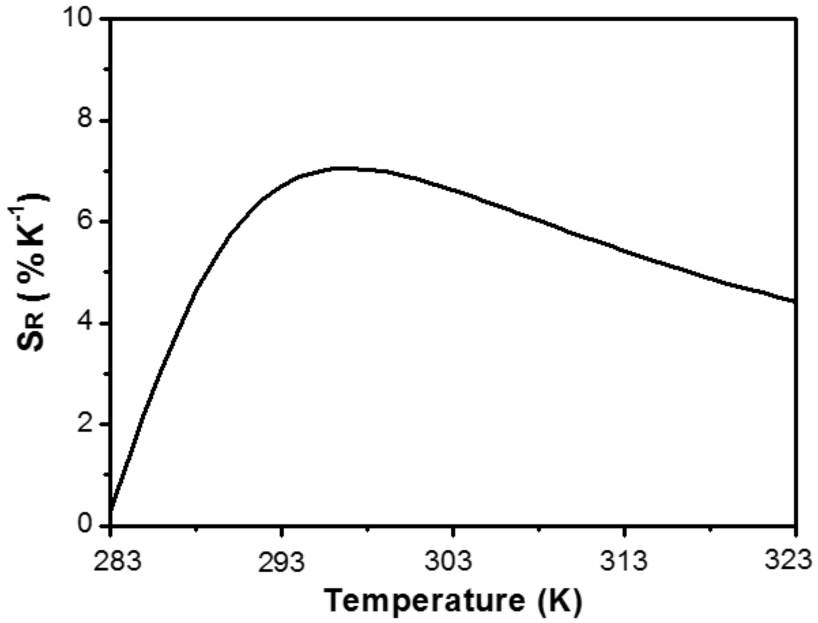

Supplementary Figure 19. The thermal sensitivity (S_R) curve of TTA-Nd-NPs. The S_R was defined by the following equation.

$$S_R = (d\ Ratio\ /\ d\ T)\ /\ Ratio \ldots \text{(equation 1)}.$$

In equation1, Ratio = $\boldsymbol{I}_{\text{TTA-UCL}} / \boldsymbol{I}_{\text{Reference}}$ = $0.00375\ T^2 - 2.12032\ T + 300.47099$, T is the temperature in Kelvin (K), and (d Ratio / d T) is a first derivative from differential analysis of the Ratio= f (T) function.

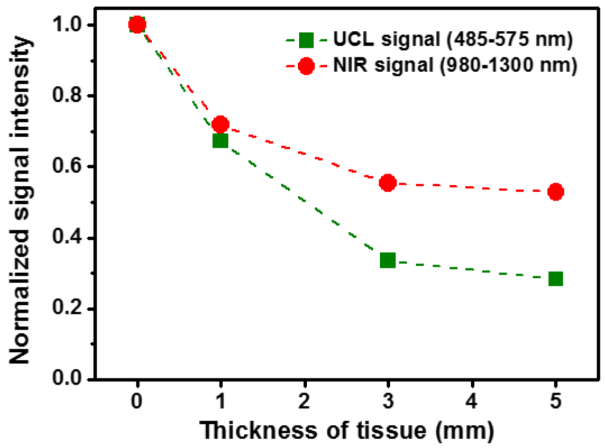

Supplementary Figure 20. Luminescence intensities of TTA-Nd-NPs under the covering of tissue phantom with thickness in the range of 0–5 mm. The emission signals were acquired in the TTA-upconversion channel I (485–575 nm, UCL signal) and Nd-NIR channel II (980–1300 nm, NIR signal) of the *in vivo* bioimaging system, respectively. The maximum intensities of the two signals were all normalized to 1.

Supplementary Note 1. The tissue could have distinct effects (absorption, scattering and reflection) on emission signals of different wavelengths. Both of the two signals emitted from UCL band and NIR band became weaker under the covering of tissues at room temperature. As the thickness of tissues increased from 1 mm to 5 mm, decreasing tendency of the NIR signal was less than that of green UCL signal, which was attributed to the mitigation of tissue effect on longer-wavelength emission.

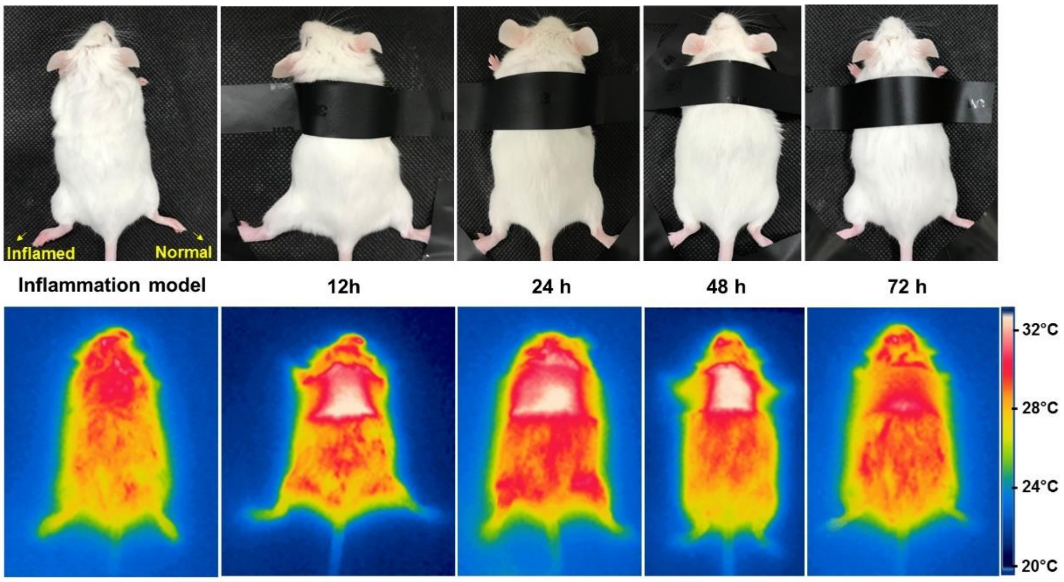

Supplementary Figure 21. The photos and thermal images of inflammation model at different time points. The left leg of a Kuming mouse was inflamed while the right leg as control was normal. The carrageenan was used to induce mild arthritis in the left leg.

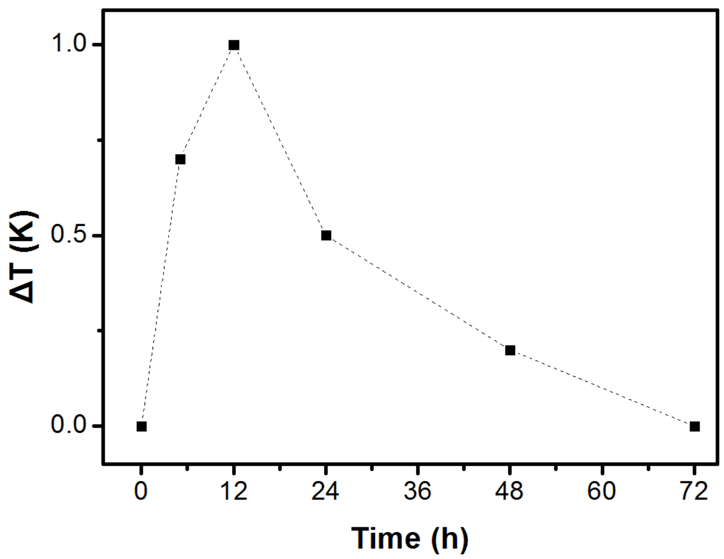

Supplementary Figure 22. The temperature deviations (ΔT) at different time points. In the inflammation model, the left leg of a Kuming mouse was inflamed while the right leg as control was normal. ΔT is the temperature deviations between two legs, namely $\Delta T = T_{left} - T_{right}$.

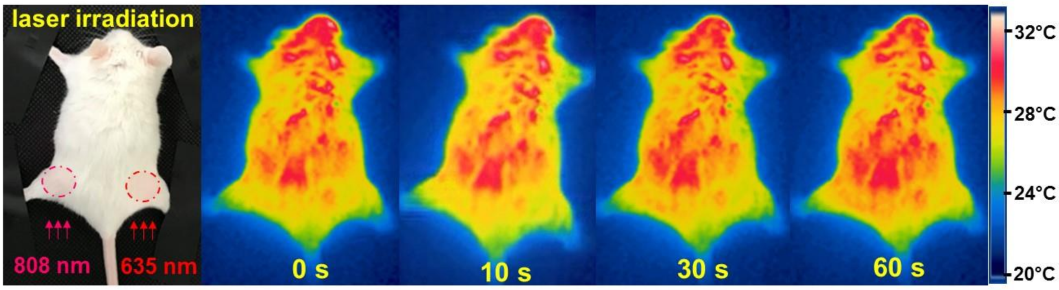

Supplementary Figure 23. The possible influence of excitation laser on thermometry. A Kunming mouse was irradiated with 635-nm and 808-nm lasers, respectively. The temperatures at different time points were recorded with an IR thermal camera. Surface temperature of the mouse showed no incensement at the first 10 s (< 0.1 K), under irradiation of whether 635-nm laser (right, 100 mW cm^{-2}) or 808-nm laser (left, 100 mW cm^{-2}). As for experimental conditions in the manuscript, the laser exposure below this level could keep influence on thermometry at a minimum.

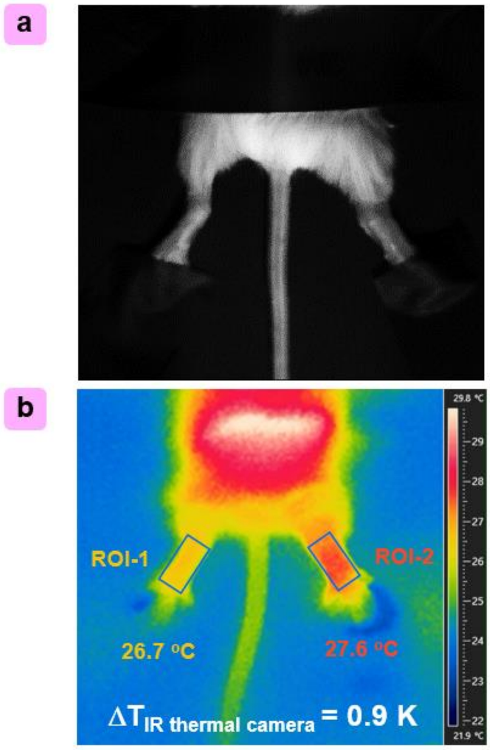

Supplementary Figure 24. The inflammation-induced temperature variation. The bright-filed image (a) showed arthritis in the swollen right leg of a Kunming mouse stimulated with carrageenan while the left leg as control was normal. The temperature distributions were measured with a thermal camera (b).

Supplementary References

[S1] a) Fu, L., Jiang, F. L., Fortin, D., Harvey, P. D. & Liu, Y. A reaction-based chromogenic and fluorescent chemodosimeter for fluoride anions. *Chem. Commun.* **47**, 5503–5505 (2011). b) Nepomnyashchii, A. B., Bröring, M., Ahrens, J. & Bard, A. J. Synthesis, photophysical, electrochemical, and electrogenerated chemiluminescence studies. Multiple sequential electron transfers in bodipy monomers, dimers, trimers, and polymer. *J. Am. Chem. Soc.* **133**, 8633–8645 (2011). c) Ahrens, J., Haberlag, B., Schejia, A., Tamm, M. & Bröring, M. Conjugated BODIPY dyemers by metathesis reactions. *Chem. Eur. J.* **20**, 2901–2912 (2014).

[S2] a) Jiang, G. C., Pichaandi, J., Johnson, N. J. J., Burke, R. D. & van Veggel, F. C. J. M. An effective polymer cross-linking strategy to obtain stable dispersions of upconverting $NaYF_4$ nanoparticles in buffers and biological growth media for biolabeling applications. *Langmuir* **28**, 3239–3247 (2012). b) Yang, T. S., Sun, Y., Liu, Q., Feng, W., Yang, P. Y. & Li, F. Y. Cubic sub-20 nm NaLuF4-based upconversion nanophosphors for high-contrast bioimaging in different animal species. *Biomaterials* **33**, 3733–3742 (2012). c) Wang, Y. F., Liu, G. Y., Sun, L. D., Xiao, J. W., Zhou, J. C. & Yan, C. H. Nd^{3+}-sensitized upconversion nanophosphors: efficient in vivo bioimaging probes with minimized heating effect. *ACS Nano* **7**, 7200–7206 (2013).

[S3] del Rosal, B., Ximendes, E., Rocha, U. & Jaque, D. In vivo luminescence nanothermometry: from materials to applications. *Adv. Opt. Mater.* **5**, 1600508 (2017).

[S4] Donner, J. S. *et al.,* Imaging of plasmonic heating in a living organism. *ACS Nano.* **7**, 8666–8672 (2013).

[S5] del Rosal, et al. Infrared-emitting QDs for thermal therapy with real-time subcutaneous temperature feedback. *Adv. Funct. Mater.* **26**, 6060–6068 (2016).

[S6] Carrasco, E. *et al.* Intratumoral thermal reading during photo-thermal therapy by multifunctional fluorescent nanoparticles. *Adv. Funct. Mater.* **25**, 615–626 (2015).

[S7] Zhu, X. J. *et al.* Temperature-feedback upconversion nanocomposite for accurate photothermal therapy at facile temperature. *Nat. Commun.* **7**, 10437 (2016).

[S8] Ximendes, E. C. *et al.* Unveiling in vivo subcutaneous thermal dynamics by infrared luminescent nanothermometers. *Nano Lett.* **16**, 1695–1703 (2016).
